# Dietary Cholesterol in the Elderly Chinese Population: An Analysis of CNHS 2010–2012

**DOI:** 10.3390/nu9090934

**Published:** 2017-08-25

**Authors:** Shao-Jie Pang, Shan-Shan Jia, Qing-Qing Man, Shuang Song, Yu-Qian Li, Peng-Kun Song, Wen-Hua Zhao, Jian Zhang

**Affiliations:** National Institute for Nutrition and Health, Chinese Center for Disease Control and Prevention, 27 Nanwei Road, Xicheng District, Beijing 100050, China; shaojiepang@126.com (S.-J.P.); jssky.good@163.com (S.-S.J.); qqm0327@163.com (Q.-Q.M.); jackie.s.song@gmail.com (S.S.); cnu_lyq@126.com (Y.-Q.L.); spk_8210@163.com (P.-K.S.); zhaowh@chinacdc.cn (W.-H.Z.)

**Keywords:** dietary cholesterol, high-quality protein, elderly population, food source

## Abstract

Dietary cholesterol intake increased dramatically over the past two decades in the elderly Chinese population. However, the nationwide dietary cholesterol intake and its related factors seldom been investigated. Based on data from 16,594 participants aged 60 years or older (49.0% male, 54.8% urban residents) from the China National Nutrition and Health Survey (CNHS) 2010–2012, we aimed to describe the intake of cholesterol and major food contributions, as well as its association with serum cholesterol level and relationship with protein intake. Mean daily cholesterol intake for all participants was 217.4 mg, the mean cholesterol intakes in urban and rural areas were 264.0 mg and 168.8 mg, respectively. Cholesterol intake levels varied by age, gender, BMI and region (*p* < 0.001). In addition, the proportion of all participants who consumed greater than 300 mg of cholesterol per day was 26.6%. Eggs, red meats, and seafood were the top three food sources and their contributions to total daily cholesterol intake were 57.7%, 24.0% and 10.9% respectively. Serum total cholesterol (TC) and low density lipoprotein cholesterol (LDL-C) were related to dietary cholesterol intake, with each 100 mg increase in dietary cholesterol intake apparently leading to a 0.035 mmol/L (*p* = 0.001) increase in serum TC and a 0.038 mmol/L (*p* < 0.001) increase in LDL-C. The partial correlation coefficients between dietary cholesterol and total protein, high-quality protein, intake of protein per kilogram body weight (BW), and high-quality protein percentage were 0.538, 0.580, 0.426, and 0.548, respectively, after adjusting for age, gender, and energy, fat and carbohydrate intakes (*p* < 0.001). In conclusion, there was a substantial urban-rural difference in cholesterol intake. Eggs and red meat were the main sources of dietary cholesterol intake. Serum TC and LDL-C were associated with dietary cholesterol and the response was linear. Dietary cholesterol intake was closely related to the intake of high-quality protein.

## 1. Introduction

China’s population is aging rapidly. It is predicted that in 2050, there will be 400 million Chinese citizens aged over 65 years old, 150 million of whom will be 80 years and over [1]. The China Health and Nutrition Survey (CHNS), a cohort study which includes one-third of the provinces in China, showed that the intake of dietary cholesterol increased dramatically over the past two decades in the elderly Chinese population [2]. Meanwhile, the Chinese population experienced a significant increase in serum cholesterol levels and the prevalence of cardiovascular-related morbidity and mortality [3,4]. Some meta-analysis studies suggest that dietary cholesterol does definitely raise serum cholesterol concentration, which is a well-established risk factor for cardiovascular disease (CVD) [5,6]. However, there is no national representative study on cholesterol intake and its association with serum cholesterol.

Furthermore, dietary cholesterol only comes from animal-based foods, which are the major source of high-quality protein, due to an ideal amino acid pattern and high bioavailability [7]. An adequate amount of high-quality protein intake is particularly important for elderly people, as it is closely associated with muscle mass and muscle function, and may delay the onset of sarcopenia [8]. WHO (World Health Organization) has recommended a daily intake of 0.8 g/kg body weight (BW) for all adults, without consideration for age [9]. In recent years, many international working groups have declared that an intake of 1.0 to 1.2 g/kg BW protein is more suitable for preserving functionality in healthy older adults [10,11,12]. It is also recommended in Chinese Dietary Reference Intakes (DRIs) that the percentage of high-quality protein should account for more than 50% of the total protein for elderly Chinese people [13].

Based on current scientific evidence and the level of dietary cholesterol intake, dietary cholesterol restriction has been dropped from the 2015 Dietary Guidelines for Americans [14]. This has led to a bitter dispute in China, since many kinds of animal-based foods, such as eggs, have been limited to elderly people due to their high cholesterol content. A report on chronic diseases and nutrition among the Chinese population showed that consumption of eggs, dairy products and aquatic products was seriously inadequate, even in the subgroup of urban elderly people with higher incomes [15]. One reason was general acceptance of the idea that strict limits on cholesterol intake are key to CVD prevention. However, with the removal of recommended cholesterol intake limitations in the United States, people have begun to question this belief. Some healthcare workers have even argued that as there is no relation between dietary cholesterol intake and incidence of cardiovascular disease, animal-based food intake should be encouraged for elderly people. There is therefore a need to know the national status of dietary cholesterol intake, its food sources, and its association with risk factors for CVD in the elderly Chinese population. Furthermore, the relationship between dietary cholesterol and protein, especially high-quality protein, is also important in order to provide evidence for suggestions concerning appropriate intake levels for animal-sourced food.

Based on data from CNHS 2010–2012, this study aims to assess the status of dietary cholesterol intake and its food sources. Furthermore, we investigate the effect of dietary cholesterol on serum cholesterol levels and the relationship between dietary cholesterol and protein intake.

## 2. Methods

### 2.1. Study Population

The survey was conducted between 2010 to 2012 by the Chinese Center for Disease Control and Prevention, from a nationally representative cross-section. Its purpose was to assess the nutrition and health status of Chinese civilians and it covered all 31 provinces, autonomous regions, and municipalities directly under the central government of China (excluding Taiwan, Hong Kong, and Macao). A stratified multistage cluster sampling method was conducted at 150 survey sites of 4 types: 34 big cities, 41 medium and small cities, 45 general rural areas, and 30 poor rural areas (these types were defined according to economic and social development characteristics). A random sample selection of 6 neighborhood committees (urban) or 6 villages (rural) was conducted. Thirty households were randomly sampled from each neighborhood committee or village, and the dietary surveys, which involved 3-day 24-h dietary records combined with food weighing, were completed by these subjects [16].

The National Institute for Nutrition and Health, Chinese Center for Disease Control and Prevention was responsible for the implementation of the national level training work (Grade 1). Provincial training plan by the project team though national organizations at the provincial level training (Grade 2). A face-to-face interview using a standard questionnaire was conducted at the homes of the participants and anthropometric measurements were taken at community health service centers, by trained staff in both cases. Weight was measured without shoes and in light clothing to the nearest 0.1 kg. Height was determined to the nearest 0.1 cm without shoes. Body mass index (BMI) was calculated as weight (kg) divided by height (m) squared [16].

Blood samples were collected from all participants after an overnight fast of at least 10 h. The samples were centrifuged at 1500 rpm for 10 min after being left standing for 30 to 60 min. The centrifuged serum samples were transported to the central laboratory of the National Institute for Nutrition and Health and stored at −80 °C. The blood collection procedure, processing, and determination were standardized. Total cholesterol (TC), low-density lipoprotein cholesterol (LDL-C) and high-density lipoprotein cholesterol (HDL-C) were measured by a Hitachi automatic biochemical analyzer with reagents from Wako Pure Chemical Industries, Ltd. (Tokyo, Japan).

In this study, we included all participants aged 60 and above who had complete demographic and dietary intake data. We excluded those with implausible energy intakes (<800 kcal/day or >4800 kcal/day for male and <500 kcal/day or >4000 kcal/day for female) and those with >2000 mg/day dietary cholesterol intake. A total of 16,594 participants (8135 male and 8459 female) were included.

The survey was conducted according to the guidelines laid out in the Declaration of Helsinki and all procedures involving human subjects were ethically approved by the Ethics Committee of the National Institute for Nutrition and Health, Chinese Center for Disease Control (2013-018). Written informed consent was obtained from all participants.

### 2.2. Dietary Data

Household food consumption data and individual dietary recall data were collected over three consecutive days. Household food consumption, including all foods and condiments, was determined by a weighing method. Individual dietary intake data were collected by asking each household member to report all food consumed (type, amounts, type of meal, and place of consumption) at home and away from home on a 3-day 24-h recall basis.

### 2.3. Assessment of Dietary Cholesterol and Protein Intake

The Chinese Food Composition Table was used to calculate individual daily intake of cholesterol for each food item in the dietary data.

Animal-based foods were divided into six categories: eggs, red meats, seafood, poultry, dairy, and other. Although dietary cholesterol restrictions have been dropped by Dietary Guidelines for Chinese Residents (2016) and the 2015 Dietary Guidelines for Americans, 300 mg/day was still used as a reference for dietary cholesterol intake in this study.

Proteins from animal-based foods and soybeans were considered to be high-quality protein. High-quality protein percentage was defined as the ratio of high-quality protein to total protein. The intake of protein per kilogram of BW was calculated as total protein intake in grams (g) divided by the BW in kilograms (kg).

### 2.4. Statistical Analysis

Data were collected using specialized software, and data analyses were performed using SAS version 9.4 (SAS Institute, Inc., Cary, NC, USA). National population census data from 2010 were used to calculate weighted dietary cholesterol, energy, protein, fat, carbohydrate, and other variables. A sampling weight was assigned to each participant based on the study design. PROC SURVEYMEANS and PROC SURVEYFREQ procedures in SAS were used to calculated mean and proportion and their 95% confident intervals (95% CI).

Differences in dietary cholesterol intake levels between genders and residences were tested with analysis of covariance (ANCOVA), with adjustments for energy, protein, fats and carbohydrates. Linear trend tests were conducted to test the trends between dietary cholesterol intake levels and age and BMI. A Cochran-Armitage trend test was conducted to test the trend between the distribution of dietary cholesterol intake levels and age. The chi-squared test was used to determine significant differences in proportions of dietary cholesterol food sources. Partial correlative analyses were used to test the correlations between dietary cholesterol and total protein, high-quality protein, protein intake per kilogram body weight, and high-quality protein percentage after adjustments for age, gender, energy, fat and carbohydrate. Linear regression models were used to study the effects of dietary cholesterol on TC, LDL-C, and HDL-C. A value of *p* < 0.05 was considered statistically significant.

## 3. Results

### 3.1. Subject Demographic Characteristics

Table 1 shows the demographics of the participants in urban and rural areas. Of the 16,594 participants, 49.0% (*n* = 8135) were male, 51.0% (*n* = 8459) were female, 54.8% (*n* = 9095) were urban residents, 45.2% (*n* = 7499) were rural residents and the mean age was 69.9 years. Intakes of total protein, high-quality protein, high-quality protein as a percentage, fat and energy were significantly higher in urban residents than in rural residents, as well as BMI, TC and LDL-C levels. Intakes of carbohydrates and protein per kilogram of BW, and HDL-C levels were lower (*p* < 0.001).

### 3.2. Daily Cholesterol Intake Level

The average daily cholesterol intake for all participants was 217.4 mg. Cholesterol intake levels were significantly higher in urban residents than in rural residents (264.0 mg vs. 168.8 mg, *p* < 0.001). There was also a significant difference in cholesterol intake between the genders (230.4 mg for male vs. 205.0 mg for female, *p* < 0.001). Cholesterol intake was higher in male while cholesterol intake per 1000 kcal was higher in female. Daily cholesterol intake decreased with age in all participants (*p* < 0.001), whereas cholesterol intake per 1000 kcal increased with age (*p* < 0.001). Daily cholesterol intake and cholesterol intake per 1000 kcal increased with BMI in all participants (*p* < 0.001, Table 2). Median values of dietary cholesterol intake levels are shown in the Table S1.

We further investigated the distribution of dietary cholesterol intake levels and found that 6.8% of participants were without almost any dietary cholesterol intake, 66.6% of participants consumed 0–300 mg/day, and 26.6% consumed in excess of 300 mg/day (Table 3). The proportion of participants with a cholesterol intake of 0 mg/day or 0–300 mg/day was significantly higher in rural residents than in urban residents (*p* < 0.001), while the proportion of those with a cholesterol intake of greater than or equal to 300 mg/day was higher in urban China (*p* < 0.001). The proportion with cholesterol intake greater than or equal to 300 mg/day was higher in male (*p* < 0.001), while the proportion that consumed 0 mg/day or 0–300 mg/day were higher in female (*p* < 0.001). The proportion of elderly who consumed greater than or equal to 300 mg/day of cholesterol decreased with age (*p* < 0.001) and the proportion who consumed 0 mg/day or 0–300 mg/day increased with age (*p* < 0.001). The mean dietary cholesterol intake among those who consumed greater than or equal to 300 mg/day was 477.3 mg; it was 135.9 mg among those who consumed 0–300 mg.

### 3.3. Food Sources of Dietary Cholesterol

**Figure 1:**
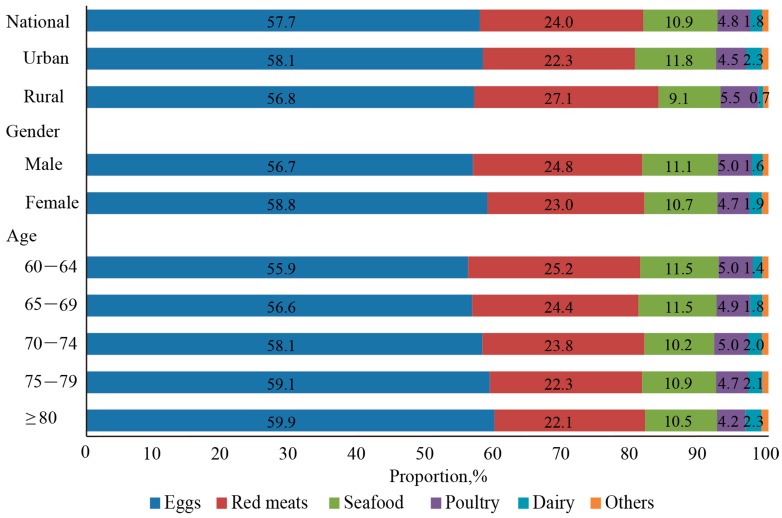
Food sources of dietary cholesterol among the Chinese elderly population by age, gender and residence.

Eggs, red meats, and seafood were the top three food sources in all participants and their contribution to total daily cholesterol intake were 57.7%, 24.0% and 10.9%, respectively (Figure 1). Cholesterol intakes from seafood (11.8% vs. 9.1%; *p* < 0.001) and dairy (2.3% vs. 0.7%; *p* < 0.001) were significantly higher in urban residents than in rural residents, while those from red meats (22.3% vs. 27.1%; *p* < 0.001) and poultry (4.5% vs. 5.5%; *p* < 0.001) were higher in rural China. There was a significant difference in cholesterol contribution rates from eggs between the genders (56.7% for male vs. 58.78% for female; *p* = 0.006). Cholesterol contribution rates from eggs (*p* = 0.003) and dairy (*p* < 0.001) increased with age in all participants, while that from red meats (*p* < 0.001) decreased.

**Figure 2:**
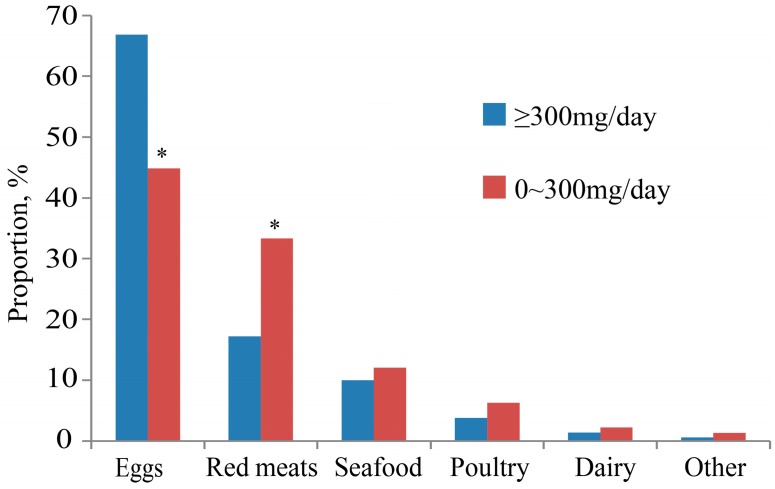
Food sources of dietary cholesterol by levels of cholesterol intake. *: Compared with the group of ≥300 mg/day, *p* < 0.001.

Comparing the food sources of the group of greater than or equal to 300 mg/day cholesterol consumption with the group of 0–300 mg/day consumption, we found that the percentage of dietary cholesterol originating from eggs in former group was higher than in the latter (67% vs. 45%; *p* = 0.002). However, the percentage of dietary cholesterol from red meats was higher in the group of 0–300 mg/day (17% vs. 33%; *p* = 0.009). No significant differences in percentage were observed in the other food sources (Figure 2).

### 3.4. The Relationship between Dietary Cholesterol and Serum Cholesterol

**Figure 3:**
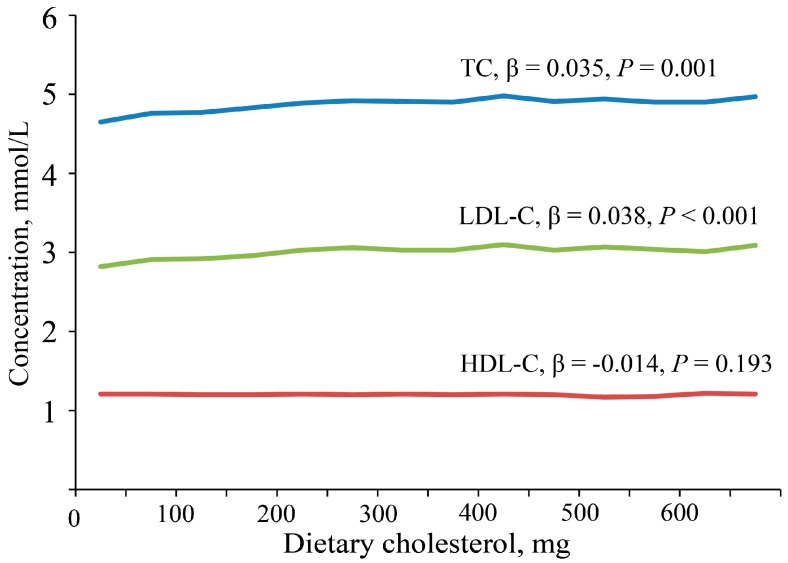
Relationship of dietary cholesterol and serum cholesterol levels. The graph represents the effect of a change in dietary cholesterol intake (per 100 mg) on serum total cholesterol (TC), low-density lipoprotein cholesterol (LDL-C), and high-density lipoprotein (HDL-C). Linear regression models were used to study the effect of dietary cholesterol on serum TC, LDL-C, and HDL-C. β represents Standardized Coefficients for per 100 mg dietary cholesterol intake.

The partial correlation coefficients between dietary cholesterol and serum TC, LDL-C, and HDL-C were 0.023 (*p* = 0.009), 0.027 (*p* = 0.002) and −0.014 (*p* = 0.101), respectively, after adjusting for age, gender, and energy, fat, and carbohydrate intakes. Serum TC and LDL-C were highest in the fourth quartile of dietary cholesterol intake and were lowest in the first quartile. There is no significant difference in serum HDL-C for dietary cholesterol intake by quartiles (Table 4). Each 100 mg increase in daily dietary cholesterol intake leads to an increase in TC by 0.035 mmol/L (*p* = 0.001), LDL-C by 0.038 mmol/L (*p* < 0.001), and a decrease in HDL-C by 0.014 mmol/L (*p* = 0.193, Figure 3).

### 3.5. The Relationship between Dietary Cholesterol and Protein Intake

The partial correlation coefficients between dietary cholesterol and total protein intake, high-quality protein intake, intake of protein per kilogram of BW and high-quality protein percentage were 0.538, 0.580, 0.426 and 0.548 after adjusting for age, gender, and energy, fat and carbohydrate intakes (*p* < 0.001).

Comparing the group with cholesterol intake greater than or equal to 300 mg/day with the group with cholesterol intake of 0–300 mg/day, the percentage of protein intake exceeding 1.0 g/kg BW/day, the proportion of high-quality protein up to 50%, and the proportion that meets both demands were 60.1% vs. 30.7%, 51.4% vs. 19.3% and 35.1% vs. 8.5% respectively (*p* < 0.001).

## 4. Discussion

In the present study, we found that dietary cholesterol intake in the elderly Chinese population varied by age, gender, BMI and region. Food sources and protein intake were different between the group with cholesterol intake greater than or equal to 300 mg/day and the group with cholesterol intake of 0–300 mg/day. Serum TC and LDL-C levels were related to dietary cholesterol intake. We also discussed the relationship between dietary cholesterol and protein intake in the elderly, which has rarely been discussed in previous research. Dietary cholesterol intake was closely related to the total protein intake, high-quality protein intake, intake of protein per kilogram of BW, and high-quality protein intake percentage.

The average dietary cholesterol intake in the elderly Chinese population was 217.4 mg/day. This was consistent with the mean dietary cholesterol intake (228 mg/day) in the global adult population in 2010 [17]. Socioeconomic status can affect daily dietary cholesterol intake. A survey conducted in a highly urbanized district of Tianjin, a big city in Northern China, indicated that the daily cholesterol intake was 430.7 mg for healthy Chinese adults [18], while another study in Guangxi, a developing province in China, showed that the mean dietary cholesterol was 199.2 mg/day in Han Chinese [19]. The present study also found that cholesterol intake levels were higher in urban residents than rural residents. Cholesterol intake was higher in males than in females for Taiwanese aged 65 and above [20], but there was no comparison for cholesterol intake per 1000 kcal between the genders. Interestingly, we found that cholesterol intake was higher in males while cholesterol intake per 1000 kcal was higher in females. Gender differences may be due to the total amount of foods intake, dietary habits, and preferences. Similarly, daily cholesterol intake decreased with age whereas cholesterol intake per 1000 kcal increased with age. Loss of appetite, dyspepsia, as well as bad dental status may be underlying reasons for why cholesterol intake decreased with age. Egg consumption increasing with age may be the reason why cholesterol intake per 1000 kcal increased with age [15].

The top three food sources of cholesterol were eggs, red meats and seafood. They contributed more than 90% of the total dietary cholesterol. The main food sources for elderly Chinese and the contribution rates were similar to those in the report of CHNS [2]. For the US and European population, the major sources of dietary cholesterol were meat, eggs and dairy products [21,22]. The percentage contributed by eggs was 57.5% for elderly Chinese compared to 25.0% for the US population [21]. This difference can be interpreted as the follows. It is widely acknowledged that eggs are highly nutritious foods, based on their high quality protein, vitamins, and minerals they provides [15]. In rural Chinese regions, especially in the west, eggs have been viewed as an affordable source of high-quality nutrition, as well as easy to cook, chew and digest for elderly people. In light of the very high cholesterol content of eggs (approximately 250 mg per egg), and the risk of CVD, there is a parallel between the risk due to eggs and that due to dietary cholesterol [23]. A meta-analysis reported that higher consumption of eggs (up to one egg per day) was not associated with increased risk of coronary heart disease or stroke [24]. The Harvard Egg Study suggested that consumption of up to one egg per day was unlikely to have substantial overall impact on the risk of cardiovascular disease among healthy men and women [25]. Recently, a cohort study indicated that higher egg consumption was associated with lower all-cause mortality [26]. However, higher dietary cholesterol and frequent egg consumption were associated with increased risk of CVD among older adults with type 2 diabetes (T2D) [27], although there was no association between egg consumption or dietary cholesterol intake and increased risk of incident T2D in healthy older adults [28]. In our study, dietary cholesterol originating from eggs in the group with cholesterol intake greater than or equal to 300 mg/day was 319.8 mg. That is equivalent to a daily intake of 1.5 eggs for these people. Existing studies indicate that 1.5 eggs per day may not cause a negative effect on health for older adults [24,25,26,28].

A meta-analysis has reported dietary cholesterol significantly increased both serum TC and LDL-C [5]. Another study concluded that each 100 mg increase in daily dietary cholesterol leads to an increase in total cholesterol by 0.056 mmol/L and HDL-C by 0.008 mmol/L [29]. In this study, we found serum total cholesterol and LDL-C were positively related to dietary cholesterol and the relationship was linear. Although the effect of dietary cholesterol on CVD risk in health adults was still not confirmed and the limit of 300 mg dietary cholesterol intake was removed from the newly revised Dietary Guidelines, it is an indisputable fact that serum cholesterol level and prevalence of cardiovascular-related morbidity and mortality are increasing rapidly in China [30]. The recommendation of consuming less than 300 mg dietary cholesterol per day for the high risk population of CVD is still being maintained in Guidelines on Prevention and Treatment of Blood Lipid Abnormality in Chinese adults (2016) [31]. Therefore, a focus on decreasing the intake of dietary cholesterol and saturated fatty acids still needs to be emphasized with a view on preventing CVD [30]. Besides, the prevalence of familial hypercholesterolemia among those aged over 50 years was high. For those people, a stricter limit on dietary cholesterol is necessary [32].

Protein is an irreplaceable nutrient for humans, and the optimization of protein intake is an important condition for preserving functionality in older persons [33]. In this study, dietary cholesterol was closely linked to total protein intake, especially high-quality protein. However, the consumption of high-quality protein was seriously inadequate among the Chinese elderly population. We found that the mean high-quality protein percentage was only 35.5% for all participants. For people living in rural areas, the protein intake per kilogram of BW was higher, whereas the high-quality protein was lower. This indicated that plant-based proteins (except for soybean) were the primary source of protein for rural elderly people. The Dietary Guidelines for Chinese Residents (2016) recommended appropriate daily intakes of 40–75 g from aquatic products, 40–75 g from meat and poultry, 40–50 g from eggs, and 300 g from milk [34]. A report on chronic diseases and nutrition among the residents of China showed that the consumption levels of dairy products, aquatic products, and eggs were seriously inadequate in the Chinese elderly population [15]. We should suggest that the public increase their intake of animal foods, especially dairy products and aquatic products, and also need to advise the public to avoid excessive dietary cholesterol intake. According to the dietary guidelines [34], daily cholesterol intake was about 400 mg. A reasonable and healthy dietary pattern, which meets the demand for high-quality protein while involving the consumption of as little dietary cholesterol as possible, should be promoted for the elderly Chinese population.

The present study was subject to several limitations. First of all, the discussion of the relationship between dietary cholesterol and serum cholesterol profile did not consider saturated fatty acid and phytosterols, both of which influence serum cholesterol. However, we adjust for age, gender, and energy, fat and carbohydrate intakes. Secondly, as with all dietary information based on dietary surveys, the accuracy of the intake estimates was limited by the accuracy of recall of the survey participants and the specificity with which the reported foods were mapped in the dietary recall records. To minimize this, all study staff members completed a strict training program that oriented them on both the aims of the study and the methodologies employed. At the training sessions, interviewers were given detailed instructions on administration of the dietary questionnaire. Thirdly, the 3-day 24-h food intake records may not reflect long-term dietary habits. Eating out is another source of potential measurement error.

## 5. Conclusions

Based on data from the China National Nutrition and Health Survey (CNHS), 2010–2012, the present study showed that average daily cholesterol intake for all participants was 217.4 mg, and dietary cholesterol intake levels were higher in urban residents and in male. Daily cholesterol intake and cholesterol intake per 1000 kcal increased with BMI. Eggs, red meats, and seafood were the top three food sources of dietary cholesterol. Serum TC and LDL-C were associated with dietary cholesterol intake. Dietary cholesterol intake was closely related to the intake of high-quality protein.

## Figures and Tables

**Table 1:** Demographic and dietary characteristics of people aged 60 and above in urban and rural areas in China.

	Nation (*n* = 16,594)	Urban (*n* = 9095)	Rural (*n* = 7499)
Age, year	69.9 (69.8–70.1)	70.0 (69.8–70.2)	69.9 (69.7–70.1) *
Male, %	49.0 (48.2–49.8)	48.4 (47.4–48.4)	49.8 (48.6–50.9)
BMI, kg/m^2^	23.7 (23.7–23.8)	24.4 (24.4–24.5)	23.0 (22.9–23.1) *
Energy, kcal	1826.1 (1816.2–1836.0)	1728.0 (1715.3–1740.6)	1928.6 (1913.1–1944.0) *
Total protein, g	54.8 (54.5–55.2)	56.5 (56.0–57.0)	53.0 (52.6–53.5) *
High-quality protein, g	21.2 (21.0–21.5)	26.0 (25.6–26.4)	16.2 (15.8–16.6) *
Protein intake per kilogram BW, g	0.95 (0.95–0.96)	0.94 (0.93, 0.95)	0.97 (0.96, 0.98) *
High-quality protein percentage, %	35.3 (35.0, 35.6)	43.0 (42.6, 43.4)	27.2 (26.8, 27.7) *
Fat, g	65.8 (65.2–66.3)	69.1 (68.3–69.8)	62.3 (61.5–63.1) *
Carbohydrate, g	255.2 (253.6–256.8)	221.7 (219.8–223.5)	290.1 (287.5–292.8) *
TC, mmol/L	4.8 (4.8–4.9)	4.9 (4.9–5.0)	4.7 (4.7–4.8) *
LDL-C, mmol/L	3.0 (3.0–3.0)	3.1 (3.0–3.1)	2.9 (2.9–2.9) *
HDL-C, mmol/L	1.2 (1.2–1.2)	1.2 (1.2–1.2)	1.2 (1.2–1.2) *

Mean values (95% confidence interval) or percentages (95% confidence interval) are shown; *: Compared with Urban, *p* < 0.001; BMI, body mass index; BW, body weight; TC, total cholesterol; LDL-C, low-density lipoprotein cholesterol; HDL-C, high-density lipoprotein cholesterol.

**Table 2:** Mean cholesterol intake by age, sex, residence and BMI.

	Dietary Cholesterol					
	Nation (*n* = 16,594)		Urban (*n* = 9095)		Rural (*n* = 7499)	
	mg	mg/1000 kcal	mg	mg/1000 kcal	mg	mg/1000 kcal
**Total**	217.4 (214.5–220.3)	125.4 (123.7–127.1)	264.0 (259.8–268.2)	158.9 (156.4–161.5)	168.8 (164.8–172.8) *	90.3 (88.1–92.6) *
**Gender**						
Male	230.4 (226.1–234.7)	122.1 (119.8–124.4)	279.5 (273.4–285.6)	155.0 (151.6–158.3)	179.7 (173.8–185.5)	88.1 (85.2–91.1)
Female	205.0 (201.0–209.1)	128.6 (126.0–131.2)	249.3 (243.5–255.0)	162.7 (158.9–166.5)	158.2 (152.8–163.7)	92.5 (89.1–95.9)
*p*-values for difference ^‡^	<0.001	<0.001	<0.001	<0.001	<0.001	<0.001
**Age**						
60–64	226.2 (221.3–231.1)	121.3 (118.7–124.0)	275.9 (268.8–283.1)	156.5 (152.5–160.5)	174.6 (168.4–180.8)	84.8 (81.7–88.0)
65–69	220.5 (214.7–226.3)	122.8 (119.6–126.1)	267.2 (259.0–275.3)	156.7 (151.9–161.4)	173.5 (165.6–181.5)	88.8 (84.7–92.9)
70–74	210.5 (203.7–217.2)	122.3 (118.4–126.2)	253.0 (244.1–261.8)	152.6 (147.4–157.9)	165.6 (155.8–175.5)	90.3 (84.9–95.7)
75–79	207.0 (198.3–215.6)	129.8 (124.3–135.4)	253.7 (241.7–265.7)	165.2 (157.2–173.2)	156.7 (144.9–168.4)	91.7 (84.9–98.6)
≥80	209.8 (199.2–220.5)	141.5 (134.4–148.6)	254.2 (239.1–269.4)	172.5 (162.6–180.8)	161.5 (147.4–175.5)	107.8 (98.3–117.2)
*p*-values for linear trend ^†^	<0.001	<0.001	<0.001	<0.001	<0.001	<0.001
**BMI**						
<18.5	188.3 (169.0–207.5)	106.9 (96.5–117.3)	243.1 (210.0–276.2)	150.7 (130.7–170.8)	165.6 (142.5–188.7)	88.2 (77.1–100.4)
18.5–24	211.4 (199.8–223.1)	119.7 (113.1–126.2)	264.1 (245.7–282.6)	157.7 (146.9–168.5)	168.1 (153.7–182.5)	88.4 (80.7–96.1)
≥24	227.3 (215.3–239.2)	130.7 (124.0–137.5)	265.3 (249.5–281.1)	157.9 (148.4–166.5)	168.8 (152.8–184.7)	89.6 (81.1–98.0)
*p*-values for linear trend ^†^	<0.001	<0.001	<0.001	<0.001	<0.001	<0.001

Mean values (95% confidence interval) are shown; *: Compared with Urban, *p* < 0.001; ^‡^: *p*-values from analysis of covariance (ANCOVA); ^†^: *p*-values from linear regression.

**Table 3:** Distribution of dietary cholesterol intake levels by age, sex and residence.

	0 mg		0–300 mg		≥300 mg	
	*n*	%	*n*	%	*n*	%
**Total**	1068	6.8 (6.6–7.0)	10,978	66.6 (66.2–67.0)	4548	26.6 (26.2–26.9)
**Region**						
Urban	233	2.6 (2.5–2.7)	5621	62.0 (61.6–62.4)	3241	35.5 (35.2–35.8)
Rural	835	11.2 (11.0–11.4)	5357	71.5 (71.1–71.9)	1307	17.3 (17.1–17.5)
*p*-values for difference ^†^		<0.001		<0.001		<0.001
**Gender**						
Male	518	6.7 (6.6–6.8)	5186	64.1 (63.7–64.5)	2431	29.2 (28.9–29.5)
Female	550	6.9 (6.7–7.1)	5792	69.0 (68.6–69.4)	2117	24.1 (23.8–24.4)
*p*-values for difference ^†^		<0.001		<0.001		<0.001
**Age**						
60–64	356	6.0 (5.9–6.1)	3935	65.8 (65.5–66.1)	1679	28.1 (27.9–28.3)
65–69	283	6.9 (6.8–7.0)	2837	65.9 (65.6–66.2)	1207	27.2 (27.0–27.4)
70–74	214	7.3 (7.2–7.4)	2147	67.5 (67.2–67.8)	856	25.2 (25.0–25.4)
75–79	139	7.9 (7.8–8.0)	1263	67.1 (66.9–67.3)	498	25.0 (24.9–25.4)
≥80	76	6.7 (6.8–6.9)	796	68.2 (67.9–68.5)	308	25.2 (25.0–25.4)
*p*-values for trend ^‡^		<0.001		<0.001		<0.001

^†^: *p*-Values from Chi-squared test; ^‡^: *p*-values from Cochran-Armitage trend test.

**Table 4:** Mean (95% CI) for dietary cholesterol intake by quartiles of individual serum cholesterol.

	Q1 (*n* = 4145)	Q2 (*n* = 4152)	Q3 (*n* = 4149)	Q4 (*n* = 4148)	*p*-Values
TC, mmol/L	4.7 (4.4–4.8)	4.8 (4.8–4.9) ^a^	4.9 (4.9–4.9) ^a,b^	4.9 (4.9–5.0) ^a,b,c^	<0.001
LDL-C, mmol/L	2.9 (2.9–2.9)	3.0 (2.9–3.0) ^a^	3.1 (3.0–3.1) ^a,b^	3.1 (3.1–3.1) ^a,b,c^	<0.001
HDL-C, mmol/L	1.2 (1.2–1.2)	1.2 (1.2–1.2)	1.2 (1.2–1.2)	1.2 (1.2–1.2)	0.633

Mean values (95% confidence interval) are shown; Q1: the first quartile; Q2: the second quartile; Q3: the third quartile; Q4: the fourth quartile; ^a^: *p* < 0.001 compared with Q1; ^b^: *p* < 0.001 compared with Q2; ^c^: *p* < 0.001 compared with Q3.

## Supplementary Materials

The following are available online at http://www.mdpi.com/2072-6643/9/9/934/s1, Table S1: Cholesterol intake levels by age, sex, residence and BMI.
