# Supplementary material for: Dietary Cholesterol in the Elderly Chinese Population: An Analysis of CNHS 2010–2012
Source: Nutrients. 2017 Aug 25;9(9):934. doi: 10.3390/nu9090934 (PMC5622694; doi:10.3390/nu9090934)
Supplement: Supplementary file 1 [file nutrients-09-00934-s001.zip › nutrients-206057-supplementary.pdf]

Supplementary Materials

Table S1. Cholesterol intake levels by age, sex, residence and BMI.

	Dietary Cholesterol					
	Nation (n = 16,594)		Urban (n = 9095)		Rural (n = 7499)	
	mg	mg/1000 kcal	mg	mg/1000 kcal	mg	mg/1000 kcal
Total	175.2 (75.6, 319.0)	99.1 (42.1, 179.5)	223.6 (117.0, 373.8)	134.6 (70.3, 217.8)	120.7 (40.8, 238.0)	63.6 (22.9, 125.1)
Gender						
Male	188.4 (83.7, 337.3)	97.7 (42.5, 174.5)	239.9 (131.4, 391.1)	132.6 (72.1, 210.9)	131.3 (44.9, 253.2)	63.3 (22.9, 124.3)
Female	162.0 (69.4, 300.2)	100.3 (41.6, 184.6)	208.4 (106.2, 356.7)	136.6 (68.7, 224.7)	113.0 (38.7, 224.6)	63.7 (22.7, 125.8)
Age						
60–64	182.1 (81.7, 323.9)	96.4 (42.2, 174.3)	234.2 (125.6, 388.9)	136.8 (71.6, 216.1)	128.7 (48.9, 245.2)	63.4 (25.3, 119.5)
65–69	177.8 (77.1, 321.9)	98.3 (42.2, 177.5)	225.4 (119.9, 372.8)	133.3 (72.2, 218.2)	121.2 (41.7, 242.8)	63.9 (22.0, 125.4)
70–74	171.6 (67.3, 314.2)	99.6 (39.9, 179.8)	213.6 (107.3, 355.3)	130.1 (66.5, 213.4)	109.0 (34.0, 235.2)	61.3 (19.5, 130.1)
75–79	160.0 (67.7, 310.0)	102.8 (41.2, 187.4)	211.2 (107.8, 362.6)	134.2 (68.0, 220.6)	103.0 (34.0, 223.0)	57.6 (19.7, 127.7)
≥80	164.6 (79.8, 310.4)	114.8 (52.5, 204.1)	211.9 (113.3, 361.4)	146.6 (78.4, 230.8)	122.0 (40.0, 229.9)	76.0 (29.1, 143.3)
BMI						
<18.5	142.0 (54.9, 266.4)	76.9 (29.9, 153.3)	195.6 (94.1, 343.6)	115.7 (57.2, 218.4)	123.6 (40.6, 233.2)	63.0 (23.8, 120.9)
18.5–24	167.6 (70.5, 309.1)	93.4 (39.0, 172.6)	219.4 (114.4, 371.3)	135.5 (69.4, 213.9)	121.3 (40.9, 239.2)	63.3 (22.8, 125.2)
≥24	187.1 (84.0, 334.0)	107.1 (47.6, 188.8)	229.6 (119.1, 377.2)	135.1 (72.2, 220.5)	120.3 (40.2, 238.8)	64.0 (22.6, 125.3)

Median values (Q1, Q3) are shown；Q1: the first quartile; Q3: the third quartile; BMI: body mass index.
